# A non-randomised feasibility study of the Rehabilitation Potential Assessment Tool (RePAT) in frail older people in the acute healthcare setting

**DOI:** 10.1186/s12877-022-03420-w

**Published:** 2022-10-07

**Authors:** Alison Cowley, Sarah E. Goldberg, Adam L. Gordon, Pip A. Logan

**Affiliations:** 1 Research and Innovation, Queens Medical Centre, Nottingham University Hospitals NHS Trust, Hucknall Road, Nottingham, NG5 1PB UK; 2 Academic Unit of Injury, Inflammation and Recovery Sciences, School of Medicine, University of Nottingham, Nottingham, UK; 3 School of Health Sciences, University of Nottingham, Nottingham, UK; 4 University Hospitals of Derby and Burton NHS Foundation Trust, Derby, UK; 5 NIHR Applied Research Collaboration East Midlands (ARC-EM), Nottingham, UK; 6 Nottingham CityCare Partnership CIC, Nottingham, UK

**Keywords:** Rehabilitation, Frail elderly, Geriatric assessment, Decision-making

## Abstract

**Background:**

Rehabilitation potential involves predicting who will benefit from rehabilitation. Decisions about rehabilitation potential must take into account personal, clinical and contextual factors, a process which is complicated in the presence of acute ill-health and frailty. This study aimed to evaluate the feasibility and acceptability of the Rehabilitation Potential Assessment Tool (RePAT) – a 15 item holistic, person-centred assessment tool and training package – in the acute hospital setting.

**Methods:**

A non-randomised feasibility study with nested semi-structured interviews explored whether RePAT was feasible and acceptable. Feasibility was tested by recruiting physiotherapy and occupational therapy participants delivering the RePAT intervention to patients alongside usual clinical care. Acceptability was tested by conducting semi-structured interviews with staff, patient and carer participants. Staff and patient characteristics were analysed using descriptive statistics. Interview data were analysed thematically. Fidelity of completed RePAT items was assessed against *a priori* criteria on how closely they matched tool guidance by two researchers. Mean values of the two scores were calculated. RePAT content was analysed and supported with verbatim quotes.

**Results:**

Six staff participants were recruited and trained. They assessed 26 patient participants using RePAT. Mean (SD) patient age was 86.16 (±6.39) years. 32% were vulnerable or mildly frail, 42% moderately frail and 26% severely or very severely frail using the Clinical Frailty Scale. Mean (SD) time to complete RePAT was 32.7 (±9.6) minutes. RePAT fidelity was good where 13 out of 15 items achieved *a priori* fidelity. RePAT was acceptable and tolerated by staff and patients. Staff participants reported RePAT enabled them to consider rehabilitation decisions in a more structured and consistent way. Patients and carer participants, although unable to comment directly on RePAT, reported being satisfied with their rehabilitation assessments which were found to embrace a person-centred approach.

**Conclusions:**

RePAT was found to be acceptable and feasible by staff, carers and patients. It allowed clinicians to make explicit their reasoning behind rehabilitation assessments and encouraged them to become more cognisant of factors which affected their clinical decision-making.

**Trial registration:**

ID ISRCTN31938453. Registered 05/10/2021.

**Supplementary Information:**

The online version contains supplementary material available at 10.1186/s12877-022-03420-w.

## Background

Rehabilitation interventions are critical to support recovery of older people living with frailty after periods of acute ill health or injury [1, 2]. Rehabilitation is a process [3] characterised by cycles of nested treatment programmes which are reviewed and refined over time. A recent consensus definition described geriatric rehabilitation as a multidimensional diagnostic and prognostic approach which aims to optimise, preserve and promote functional reserve, capacity, well-being and social participation [4]. Healthcare professionals are frequently required to make recommendations about patients’ potential to respond to and benefit from rehabilitation. In acute hospitals they may have limited time to make assessments which take into account the complexities of frailty and superimposed ill-health. Decisions about rehabilitation potential can influence whether patients can access rehabilitation services [5, 6]. Frailty may effect an individual’s rehabilitation potential [7]. Frailty is a concept used by clinicians which describes a state of increased vulnerability to poor resolution of homeostasis after a stressor event [8] and is associated with adverse clinical outcomes and patient experiences [9, 10].

A recent systematic mapping review found [11] found that concepts of rehabilitation potential in older people could encompass prognostication (a prediction of what could be achieved with rehabilitation programmes) and outcome measurement (a retrospective understanding of what had been achieved) but that assessments tend to be based upon a snapshot of older people’s abilities rather than taking account of the dynamic nature of frailty and rehabilitation practice. A qualitative focus group study found that rehabilitation potential assessments in the acute setting coalesced around three clinical questions – “will it work?”, “is it wanted?” and “is it available?” [12]; but there was a paucity of structured approaches and frameworks that could support clinical decision-making. A person-centred, multi-disciplinary and holistic approach to rehabilitation potential assessments was recommended, with some similarities to Comprehensive Geriatric Assessment models of care [13]. Person-centred care focuses on the needs, preferences and values of individuals in order to guide and inform clinical decisions [14].

Despite this, there is no universally agreed, systematically assessed or operationalised model or clinical guideline that helps clinicians make consistent, transparent, patient-centred and evidence-based decisions about rehabilitation potential. Adhering to the Medical Research Council Framework for developing and evaluating complex interventions [15, 16] the Rehabilitation Potential Assessment Tool (RePAT), a 15 item assessment tool and training package which emphasise person-centred holistic approaches was developed [17].

The aim of this study was to evaluate whether the RePAT intervention was feasible in the acute hospital setting and whether it was acceptable to healthcare professionals, older people living with frailty and their care givers.

## Methods

This study was reported in accordance with CONSORT 2010 guidelines for randomised feasibility and pilot studies [18] and intervention described according to the Template for Intervention Description and Replication (TiDieR) guidelines [19]. The study was reviewed by the Yorkshire & The Humber – Bradford Leeds Research Ethics Committee on 21^st^ November 2017 and a favourable opinion was given on the 3^rd^ January 2018 (17/YH/0356 IRAS project ID 227288). The study was conducted according to the Declaration of Helsinki. Written informed consent was obtained from all participants prior to participation and they were all assured that they could withdraw their consent at any time without consequence.

### Study design and setting

A non-randomised controlled, single-centre, feasibility trial, with an embedded qualitative component was conducted. Physiotherapists and occupational therapists working on geriatric medicine wards in a large acute teaching hospital were recruited and trained to use the RePAT intervention between March and June 2019. Patients receiving care on the wards were recruited then assessed with RePAT. These clinical areas assess and treat older people with acute medical problems, most of whom have complex care needs, frailty and experience functional deterioration associated with their admission.

*A priori* criteria of success for feasibility were: (1) Five staff members recruited within one month: (2) Twenty-five patients recruited within two months: (3) Intervention delivered to 25 participants: (4) Fidelity of item completion achieved on the RePAT intervention at 80%. Acceptability was explored through semi-structrued interviews.

### Staff Participants

A convenience sample of occupational therapists and physiotherapists working on geriatric medicine wards were invited to take part using posters displayed in staff areas (from a population of 20 therapists). The sample consisted of clinicians with varying levels of clinical experience in care of older people and seniority. Informed written consent was obtained prior to participation. A sample of five staff participants and 25 patient participants was proposed to generate meaningful inferences from the data and as deemed feasible within the study timeline.

### Patient and family participants

Potential patient participants were screened by the staff participants and researcher against inclusion criteria (Table 1). Staff participants made the initial approach then a researcher undertook formal informed written consent. Mental capacity was assessed in line with the Mental Capacity Act [20] and cognitive abilities were assessed using standardised measures such as the Mini-mental state examination (MMSE) and the Abbreviated Mental Test (AMT). For potential patient participants lacking capacity, an appropriate consultee was approached to complete a consultee declaration and consent form. Family participants who took part in the semi-structured interviews provided informed written consent.

**Table 1:** Participant eligibility criteria

	Inclusion criteria	Exclusion criteria
**Staff participants**	Physiotherapists or occupational therapists working in the acute care setting with older people living with frailty, specifically carrying out rehabilitation assessments or programmes of rehabilitation.	Physiotherapists or occupational therapists involved in research studies exploring or testing rehabilitation potential, rehabilitation assessments or rehabilitation models of care for older people living with frailty. Staff participants working in specialist stroke, end of life or fracture services.
**Patient participants**	Participants identified as frail using the Clinical Frailty Scale by staff delivering routine clinical care and those in receipt of rehabilitation assessments or programmes of rehabilitation Participants able to give informed consent or if assessed and deemed to lack capacity consultee agreement from care givers or family member or appropriate consultee.	Participants in receipt of specialist stroke rehabilitation, specialist fracture care, specialist end of life or with a terminal diagnosis. Participants with advanced care plans or directives, which stated that they did not wish to take part in research studies. Patient participants found to lack capacity for whom an appropriate consultee could not be identified.
**Family/carer participants**	Carers or family members of patient participants in receipt of rehabilitation assessments or rehabilitation programmes.	

### The RePAT intervention

RePAT comprised 15 items/questions relating to components of rehabilitation potential (Supplementary file 1) where clinicians were asked to document their assessment findings and clinical reasoning. A more detailed description of the RePAT intervention development can be found in Cowley et al [17]. Staff participants attended a 60 minute training session on RePAT which included: background to the development of RePAT, patient participant recruitment process, mental capacity, data collection and RePAT completion using a clinical vignette. Opportunities for discussion and reflection were provided.

### Data collection

Staff participants completed a demographic information sheet detailing their profession, job banding according to Agenda for Change (AFC) [21] and clinical experience in geriatrics.

Patient characteristics, that may inform predictions of rehabilitation potential [11], were identified from medical notes and electronic hospital records by the research team. These included: age, gender, ethnicity, residential and cohabitation status, co-morbidities, medications, cognition, frailty status, pre-admission mobility, ADL abilities and reasons for admission to acute care. Admitted complaints were classified according to the International Classification of Disease 11 (ICD-11), a globally standardised method for reporting and classifying diagnostic health information [22]. This provided a description of the patient population included in the study. Assessments completed as part of usual clinical care, number of prescribed regular medications, cohabitation status and pre-morbid levels of mobility and independence, number of types of multi-disciplinary team (MDT) assessments, goals, outcome measures and types of rehabilitation planned (or not) were recorded on the case report form. The Charlson Co-Morbidity Index (CCI) was chosen to quantify underlying medical conditions in patient participants [23]. The Clinical Frailty Scale is a pictorial representation used to stratify individuals with frailty based on their level of vulnerability [24] using a nine point scale where 1 is ‘very fit’ up to 9 ‘terminally ill’.

Staff participants were asked to assess patient participants’ as per usual clinical care and then re-assess using the RePAT intervention. A researcher met with staff participants after they had completed their first RePAT assessment, to provide support and mentorship and clarifying any questions on the intervention. The completed RePAT forms were then collected and data were transferred onto an Excel spreadsheet for fidelity assessment and data analysis.

### Intervention fidelity

Fidelity considers if the intervention was delivered as intended [25, 26] and is central in understanding if staff participants were able to deliver the intervention effectively [27]. *A priori* fidelity assessment criteria were developed which considered how closely the content of the completed RePAT items matched the guidance, outlined in the tool and in the training. Each item was rated as either having 100%, 50% or 0% fidelity (Table 2) on how closely they matched tool guidance by two researchers. Mean values of the two scores were calculated. Although there are no standardized measures of intervention fidelity, an 80% agreement rate has been suggested as an acceptable measure [28, 29].

**Table 2:** Fidelity criteria

Fidelity level	Description
100%	Fully meets criteria
50%	Meets some of the criteria
0%	Does not meet criteria or empty cell

### Data analysis

Patient and staff participant characteristics and usual care descriptors were analysed using descriptive statistics including means, standard deviations, range for quantitative variables and counts for categorical data using STATA15. Free text data from completed RePAT interventions were supported with verbatim quotes for each item alongside fidelity ratings described above.

Patient and staff participant interviews were thematically analysed separately using six stages of analysis which included: data familiarisation, coding, development of themes, reviewing themes, defining and naming themes and reporting [30]. Data was analysed by AC and members of the study team ALG, PAL, SG and MK. Reflective diaries were kept and incorporated into the analytical framework. This approach provided a rich and detailed account of participants’ experiences of using or being assessed with the RePAT.

## Results

Three out of four *a priori* criteria for success were achieved (Table 3).

**Table 3:** Criteria for success

Criteria	Outcome
Five staff participants recruited within one month	Achieved
25 patients recruited within two months	Achieved
Intervention delivered to 25 participants	Achieved
Fidelity of intervention at 80% for all items	Not achieved

### Patient participant recruitment and retention

**Fig. 1:**
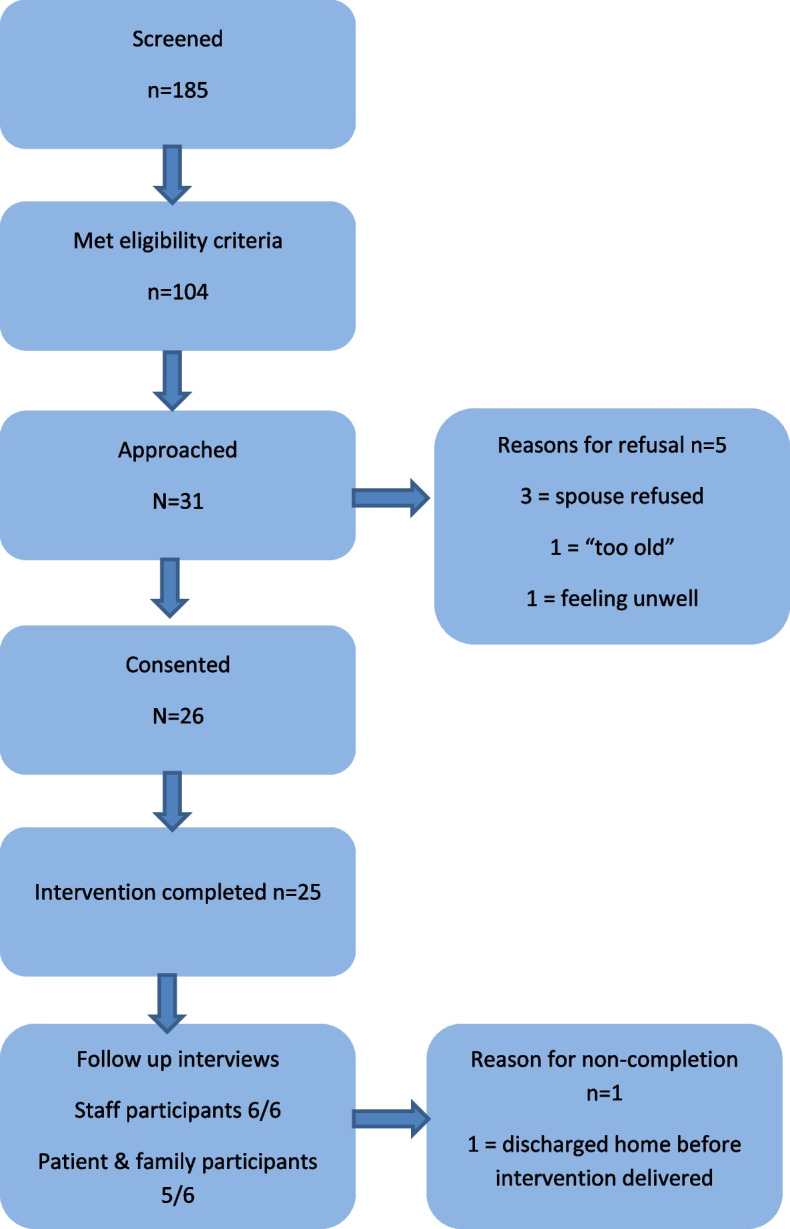
Patient and family participants flow through the study

A total of 185 patient participants were screened and 104 met the inclusion criteria. Thirty one patients were then approached. Other potential participants were not approached due to the patients being acutely unwell or absent from the ward area and the feasibility study reaching its recruitment target. Patent and family member participant flow through the study is summarised in Fig. 1.

### Staff participant demographics

Staff participant demographics are displayed in Table 4. Initially five staff participants were recruited to the study. Due to clinical time restrictions, one participant (SP 3) was unable to complete five patient assessments so an additional staff participant was recruited. In the United Kingdom, physiotherapists and occupational therapy job roles are classified according to Agenda for Change with band 5 being the most junior staff members.

**Table 4:** Staff participant demographics

**Profession**	
Physiotherapist	3
Occupational therapist	3
**Banding**	**UK AFC **[21]
Band 5	4
Band 6	2
**Highest qualification**	
BSc	5
BSc (Hons)	1
**Time working on acute geriatric wards (months)**	
Mean ± SD	18 ± 32.24
Range	4 – 84
**Rehabilitation experience with older people (months)**	
Mean ± SD	33.17 ± 28.4
Range	7-84
**Time qualified (months)**	
Mean ± SD	50 ± 50.36
Range	10-144
**Sample size**	**6**

### Patient participant demographics

Twenty-six patients were recruited to the study. Patient participant characteristics were reported for 25 participants (Table 5), accounting for the one participant who was not assessed due to time restrictions.

**Table 5:** Patient participant demographics

**Age**	**Mean, SD, range**
Years	86.16 (± 6.39)71-107
**Gender**	**N, %**
Male	16 (64%)
Female	9 (36%)
**Ethnicity**	**N, %**
White	24 (96%)
Mixed Race	1 (4%)
**Pre-admission residential status**	**N, %**
Home with no formal care support	15 (60%)
Home with formal care support	7 (28%)
Assisted living	2 (8%)
24-hour live-in carer	1 (4%)
**Preadmission mobility status**	**N, %**
Independent with no aid	2 (8%)
Independent with stick	3 (52%)
Independent with frame	9 (36%)
Assistance of one plus aid	1 (4%)
**Cognitive screening tools**	**N, %, mean score, SD**
Abbreviated AMT	9 (37.5%) 3.11 (± 1.54)
AMT	14 (58.3%) 6.86 (± 3.1)
MMSE	1 (4.17%) 18
**Clinical Frailty Scale**	**N,%**
Well	1 (5.3%)
Vulnerable	3 (15.8%)
Mildly frail	2 (11%)
Moderately frail	8 (42%)
Severely frail	4 (21%)
Very severely frail	1 (5.3%)

On admission patient participants had a mean of 6.2 comorbidities (± 2.73) and a mean CCI of 3.44 (± 2.12). The most common comorbidities were hypertension, cerebrovascular disease, dementia, kidney disease and chronic obstructive pulmonary disease. All participants took regular prescribed medications with a mean number of 7.54 (± 3.11). Patients were admitted with a mean of 2.12 ICD-11 codes (±0.73). The most frequent were falls (26.41%), mobility difficulties (17%) and disorientation (7.55%). Cognitive screening occurred in 96% of participants with the AMT and abbreviated AMT being used in 37.5% and 58.3% of the sample. The sample comprised of patients with mild to moderate degrees of cognitive impairment. Nineteen (76%) participants were assessed using the Clinical Frailty Scale on admission [24], where 42% were classified as moderately frail and 21% as severely frail. No other frailty assessment tools were reported.

Staff participants set and documented goals with 32% (n=8/25) of patient participants and involved family members in assessments or rehabilitation plans in 56% (14/25) of participants. Aims of rehabilitation documented in usual care were to: “increase indoors and outdoors mobility”, “improve confidence in everyday functioning”, “increase independence with Activities of Daily Living” or not specified in usual care.

### The novel intervention – RePAT

Twenty five patient participants were assessed with RePAT. RePAT assessments were fully completed with no missing data. The mean time to complete the RePAT was 32.7 minutes (range 15 – 60 minutes). The results for each RePAT item are displayed in Table 6 alongside verbatim quotes from the completed RePAT tools.

**Table 6:** RePAT intervention results

Item	Supporting Evidence	Mean fidelity score (%)
Are there any underlying physical issues which may affect or interfere with rehabilitation?	*“Atrial fibrillation, previous stroke (left MCA infarct), hypertension, and poor vision. May cause difficulties in different environments.” SP1/PP19* *“Hypertension, postural drop currently being investigated so patients’ mobility should improve once management is optimised.” SP4/PP6*	100
Are there any unresolved physical issues which may affect or interfere with rehabilitation?	*“Resolving urinary sepsis, functional decline following infection. CT lumbar spine showed L5/4/3 endplate collapse. Pain in lumbar spine limits activities.” SP1/PP25* *“Nil of note, medically fit for discharge, no medical follow up.” SP5/PP7*	100
Are there any underlying psychological issues which may affect an individual’s motivation or participation with rehabilitation?	*“Mild hypoxic brain injury, patient requires clear step-by-step instructions and struggles with memory.” SP6/PP26* *“Nil, very keen to progress.” SP3/PP3*	100
Are there any unresolved psychological issues which may affect an individual’s motivation or participation with rehabilitation?	*“Hallucinations settled during admission and had increased mood within inpatient progression.” SP3/PP1* *“Nil, very motivated to participate in rehabilitation, nil evidence of delirium.” SP3/PP2*	100
Has the individual been able to demonstrate participation in the rehabilitation assessment or rehabilitation programme during this current episode of care?	*“Patient was motivated and engaged in all Occupational Therapy [OT] and physio assessments.” SP2/PP14* *“Patient has been keen to participate, clear instructions required due to confusion. Pain has been limiting factor.” SP6/PP26*	100
What are the individual’s current functional abilities and levels of independence?	*“Independently mobile with wheeled frame, independent changing stoma. Assistance with washing and dressing, independent with all transfers.” SP4/PP13* *“At best had assistance of 2 to mobilise 10m. Mostly handled 2 person step round transfer.” SP3/PP4*	88.2
What were the individual’s pre-morbid (pre-admission) functional abilities and levels of independence?	*“Pre-admission (3/52) independently mobile, unlimited exercise tolerance and driving. But 3/52 prior to admission a slow decline due to pain and difficulty with transfers.” SP3/PP3* *“Lives alone, no care package. Has cleaner and support from family. Independent with shopping, meals and personal care. Mobilises with walking stick indoors and electric scooter outdoors.” SP4/ PP6*	88.2
Do you have a thorough understanding of an individual’s environment in their usual place of residence?	*“Yes, through discussion with patient.” SP1/PP19* *“Patient lives in bungalow, 1 step access, all level inside, riser recliner chair, Mowbray toilet frame and double divan bed.” SP2/PP17*	88.2
What support does the individual require to make decisions about their future?	*“Wife supports with all ADL’s and decisions. Lacks capacity however, this is variable due to resolving delirium and UTIs.” SP5/PP15* *“No concerns regarding capacity, patient has support from family.” SP6/PP23*	73.5
Has the individual been asked, “What’s important to me?”	*“I just want to go home [patient]. Go home with the right support and equipment so I can manage as much as I can on my own [husband].” SP5/PP8* *“To return to previous baseline function so she is able to toilet independently.” SP3/PP2*	97
Have goals been set and agreed which are SMART? (Short, Medium, Long Term)	*“Short – transfer with wheeled frame in 2 days. Medium – go to rehab and mobilise with wheeled frame in 2 weeks. Long – return home independently mobile with wheeled frame in 3 weeks.” SP4/PP1* *“To return to independence with personal care and cooking within 4 weeks.”* SP6/PP24	55.9
Has the multi-disciplinary team been involved in the assessment or decision-making process?	*“Review by OT, social services, medical review, liaised with daughter.” SP4/PP13* *“Nurses report patient is becoming reliant and refusing certain medical interventions but keen for rehab. Integrated Discharge Team to review.” SP4/PP6*	94
Has the individual's rehabilitation potential been assessed over multiple time points?	*“Patient assessed every morning with nursing staff, OT and physio reviewed patient daily whilst on ward.” SP2/PP14* *“Long admission of 41 days and has reduced motivation, fluctuating cognitive states have been seen.” SP3/PP4*	88.2
Is the proposed rehabilitation programme likely to be effective?	*“Given the right amount motivation and improved medical status I think residential rehabilitation would benefit the patient.” SP4/PP5* *“Patient has already improved whilst on the ward and this is likely to continue at home with support.” SP6/PP23*	94
Overall impression	*“Patient is likely to regain independence and confidence with the reablement team.” SP2/PP14* *“Patient has demonstrated determination with little encouragement needed to participate in therapy. Is dependent on medication timing and mental state, however has progressed with each session. I feel that will improve once back at home in familiar environment and once UTI’s and delirium have settled.” SP5/PP15*	88.2

Staff participants are referred to as SP plus their study number (for example SP1) and patient participants as PP plus their study number (for example PP1)

### Staff participant interviews

Six staff participants took part in the semi-structured interviews, which lasted between 20 and 41 minutes (mean 31 minutes). Themes were categorised as: enhanced clinical reasoning in a pressured environment, embracing complexity with a holistic approach, patient-centred approach to decision-making, feedback loop and implementation into future practice.

1. Enhanced clinical reasoning in a pressured environment

Participants reported that RePAT had had a positive effect on their clinical practice, providing structure for their clinical reasoning and decision-making.

*“I found it useful because it made me think, the clinical reasoning behind why we did it…it does actually make you reflect on what you have done and why you have done it.“* [Staff participant two]

Participants spoke of how the rapid turnover of patients in the acute hospital setting and pressures to free up beds meant that sometimes their preferred way of working was comprised. RePAT provided a structure and process to make complex decisions quickly, systematically and robustly.

*“I think the difficulties are the time restraints which stop you. You don’t always have as much time to dig down into the pre-admission issues from the physical and the cognitive point of view. I think [RepAT] helped to just go back and gives you a bit more of holistic goals and clear understanding of the patient.”* [Staff participant one]

2. Embracing complexity with a holistic approach

Participants expressed that the complexity and fluctuation in the performance of their patients meant that decisions were often complex and influenced by day-to-day processes and non-clinical factors. RePAT helped participants to consider a wide range of factors about an individual’s rehabilitation potential in a robust and systematic fashion. This was said to lead to improved inter-disciplinary working and a more holistic approach to patient care.

*“It was certainly useful, having it all together because then everyone could see that this is what evidence you’re basing your decisions on.”* [Staff participant six]

RePAT supported a holistic approach by covering a range of physical, psychological, environmental and participatory items. Although these were included in their routine clinical practice, RePAT required them to explore these in greater depth and the impact these may have had on rehabilitation potential.

*“They come into hospital with a new acute issue, so, looking at their baseline function, with all the comorbidities. I probably more put it to the back of my mind unless there's something quite significant that is going to have an impact. So if there's a musculoskeletal issue, delirium, nutrition or they're not eating or drinking. … then you always have to think about how that's going to influence your treatment on that day and in the future…and then we'd just have to think how they are going to recover from that.”* [Staff participant five]

For some participants, this led to a deeper understanding of delirium, mood and cognition and the impact these had on abilities and performance. Whilst routine assessments were found to categorise patients into potential for improvement or not, or matched patients services, RePAT allowed clinicians to take account of the complexity of their patients.

*“Things that are unresolved, psychological issues, delirium is such a big issue. I think in the frail population as well, so just bringing it up it makes you think, actually is this resolving, what is the pattern, has this happened before, is this from a previous admission or are we likely to get better and perhaps think about rehab, slightly later down the line?”* [Staff participant three]

Some participants wanted a binary decision on rehabilitation potential. However, they contradicted themselves by stating that a tick box or yes/no approach to rehabilitation potential was not helpful in addressing the complex needs of their patients. Free text boxes presented in the RePAT allowed them to delve into the nuances and fluctuations associated with frailty.

*“I know with a lot of other decision-making tools you have might yes/ no questions, and then you kind of how many yes’s how many no’s but actually people aren’t that straight forward.”* [Staff participant six]

Although participants stated that they routinely considered rehabilitation potential over multiple time points within a patient’s stay, they admitted that this was often assumed rather than formally assessed. RePAT prompted them to ensure that they adopted an iterative approach to considering rehabilitation potential, which took into account fluctuations in health, performance and participation.

*“I got down to a certain part so ‘had they been assessed at multiple points?’ I’ve thought no.”* [Staff participant three]

3. Patient-centred approach to decision-making

Participants reported that RePAT enhanced their ability to use a patient-centred approach to decision-making and understand patient and carer wishes. They stated that they and members of the MDT frequently assumed that they knew what a patient or carer wanted to achieve but did not fully understand this until they were prompted to ask “What is important to you?” This item highlighted that they frequently considered what was important to them and their organisation rather than patient needs and wishes.

*“Putting the patient at the front of the plan going into MDT discussions. It does sometimes feel like the patient doesn’t have a voice. [RePAT] helps to keep the patient at the forefront.”* [Staff participant one]

This item was also used to enhance their goal setting and focus on what was relevant to the patient and carer. However, goal setting was frequently cited not to be prioritised in clinical practice due to acute setting pressures, subsequently, many participants felt deskilled in goal setting practice.

*“Not enough of us [goal set]. In our medical notes, we might write something, it might be a goal, but it’s not smart. Mainly never agreed with the patient, doesn’t have a timeframe…but here it’s not such a priority for people whereas actually that is a massive part of your rehab potential prediction. If you think that we have set these short term goals have we achieved them, yes, right you will very likely achieve this. If you have not achieved any of our goals within two or three days actually, rehab potential wise, what are we aiming for, what is going to be realistic?”* [Staff participant three]

4. Feedback loop

Participants spoke of observing rehabilitation response and progress during a patient’s hospital stay but feedback on future progression was largely absent. They spoke of being unaware of the outcomes of proposed programmes of rehabilitation and therefore were unable to judge if their clinical decisions were accurate or successful.

*“We don’t know what happened to them because we don’t get the feedback…if the rehab unit they went to think they are unsuitable.”* [Staff participant three]

Staff participants attributed this lack of feedback to a number of factors. This included the lack of shared IT systems across organisations, time pressures which meant that progress was not explored and the lack of emphasis on rehabilitation outcomes.

*“Are we making the right decision, are we making the right discharge pathways for these patients? I do think we need a piece of work on this.”* [Staff participant one]

5. Implementation into future practice

Participants positively evaluated RePAT training, finding the clinical vignettes helpful in terms of theory and practice. They referred back to the training during the early stages of using RePAT and reported that the speed and ease with which they completed RePAT increased. They suggested that supplemental online training or peer mentoring would be helpful for future trials.

*“The training tool was good and after I did that I thought, yes, right, I can definitely do this but then when I came to do it on a patient, the first two it took me a lot longer. But I think you still need that case study to go through as an example. I can’t imagine trying to do it without a case study,.”* [Staff participant three]

Participants reported that they obtained information from multiple sources such as existing clinical notes (paper-based and electronic), other MDT members, patients and carers to complete RePAT.

*“I just went to my normal routine of getting as much information from everywhere. So it could be from the notes, it could be from the family, it could be from the computer system.”* [Staff participant five]

During this feasibility study, RePAT was used alongside routine clinical practice and hence in addition to completing usual assessments and supporting paperwork. Participants agreed that RePAT could replace their usual practice for rehabilitation assessments and decisions on rehabilitation potential and be integrated electronically.

### Patient and family participant interviews

Three patient-participants took part in the interviews and a husband of a patient participant with a diagnosed dementia (family participant one). Themes that emerged were: who was that, what’s important to me, family involvement and communication, and I just want to go home.

1. Who was that?

Patient participants were unable to distinguish RePAT from routine care. Participants stated that due to the nature of the hospital environment, they were frequently unable to identify the role of different professionals.

*“There are lots of different people, that have talked to you in hospital and it’s muddling… I recognise them, but you forget their names.”* [Patient participant 24]

Participants reported being assessed over multiple times and in some cases, were able to outline certain assessments. The roles of allied health professionals were less well defined. Participant 24 explains an assessment by a physiotherapist.

*“I think she pressed my feet against… they asked me about my toilet at home, they are going to give me a frame to go round it because my toilet is too low, so I can lift myself up.”* [Patient participant 24]

2. What’s important to me?

Patient participants reported that they had been asked to state what was important to them and what they would like to achieve.

*“I said to get fit enough to be able to go home. We have got a beautiful garden, we have just had it re-landscaped but there is a few bits and pieces that we want doing. I would love to get out there and do a bit..”* [Patient participant 18]

In the hospital environment, choices about acute care were nuanced by the belief that they were in *“the right place to be ill” [Patient participant 20]* and that healthcare professionals knew what was best for them. This meant that they often deferred power and choices to professionals or family members.

3. Family involvement and communication

Participants spoke of the importance of involving family members in their care, decision-making and providing information to healthcare professionals.

*“My daughter wanted them to try walking me upstairs…I need to do them to go home, which they did with her. They said it was fine.”* [Patient participant 20]

However, this was not without challenges. A family member who was the sole carer for a participant with severe dementia, spoke of feeling helpless and at odds with healthcare professionals in the care and future plans for his wife.

*“They tried her on a rotunda this morning. I said a darn sight better if you just lifted her up and moved her. All this walking, leaning, spinning around. That is not good. They explained the principle of it and I’ve got one delivered to me at home but I can’t see me using it.”* [Family participant one]

He had expressed a desire for a place at a certain rehabilitation facility and remained unclear why his wife was not suitable.

4. I just want to go home

Participants spoke of the desire to return to their usual place of residence and felt their ill health and the hospital environment impacted adversely on their functional abilities.

*“It’s a job to stand up. I don’t know why they think this flooring is marvelous, but your feet are slipping all the time. I have one of those wheels there [points to a three-wheeled walker], I’ve never used them with before and that was foreign to me.”* [Patient participant 20]

They expressed the belief that once they returned home, they would cope and perform better. This was strongly voiced by the family participant of a patient with dementia.

*“I just want her home and will sort the rest out from there.”* [Family participant one]

Participants were unable to provide specific recommendations on how RePAT could be improved or implemented into practice, but at the heart of their talk was the need for clear communication, partnership working and embracing their needs, wants and wishes.

## Discussion

This study found that it was feasible and acceptable to deliver and incorporate RePAT into usual care in an acute healthcare setting. Staff participants were recruited, trained and delivered RePAT with a high level of fidelity. They reported that RePAT provided structure for their rehabilitation potential decision-making. Eligible patient participants were identified and recruited into the study which included those with cognitive impairment. Patient, carer and family participants were unable to isolate experiences of RePAT from usual care but the majority reported positive experiences of RePAT and their care.

Recruitment in the acute hospital setting has found to be fraught with challenges associated with a busy, confusing environment and the onset of acute ill health [31]. One hundred and eight-five patients were eligible to enter the study, 31 were approached and 26 participated. Patient participants were acutely unwell, frail and had complex health and social care needs. This sample was similar to those in other studies [32, 33] but older people with complex needs are often deemed too complicated or too frail to recruit [34].

Staff participants with a range of clinical experience found RePAT useful in their clinical decision-making but the sample predominantly consisted of clinicians working at more junior levels of experience and expertise. Although clinical experience has been traditionally associated with years of experience, expertise is influenced more by clinicians’ experience of health-related quality of life outcomes and patient experience [35].

This study found that clinicians adopted unstructured and intuitive approaches to rehabilitation decision making prior to using RePAT. Participants frequently recognised a mismatch between their stated objective of conducting iterative, multi-disciplinary, person-centred assessments and the reality of the much more minimal assessments that they were able to conduct in their real-world settings. Unstructured approaches to decision-making increases variability in clinical practice [36] and may lead to poorer patient outcomes. The RePAT intervention provided a structured approach to rehabilitation potential decision-making which allowed clinicians to consider multiple clinical and patient level factors such as pre-morbid abilities, underlying conditions, motivational factors and patient goals alongside contextual factors such as rehabilitation resource availability, type, dose and intensity. Rather than condensing assessments of rehabilitation potential into a simplistic binary form, which fail to take into account the fluctuations and uncertainties of frailty presentations [12], a more nuanced approach was adopted. RePAT used a predictive or conditional model of clinical reasoning where clinicians embrace the complexities of prognostication with clinical presentation, outcomes, timeframes and demands that biological, psychological, environmental and personal factors are taken into account [37, 38]. Similar approaches have been adopted by the World Health Organization to reduce mortality and morbidity in surgical safety using a structured checklist approach [39]. Staff participants reported that they seldom received feedback on the outcome of their clinical decisions on rehabilitation potential and rehabilitation assessments. Feedback on outcomes of previous decisions allows clinicians to become aware of their limitations and abilities in clinical decision-making [40]. Although RePAT did not provide participants with feedback on the success of their rehabilitation potential assessments, future implementation of the tool may provide opportunities for professional development through guided reflection with colleagues [41] across health and social care.

The RePAT intervention included theoretical and practical components. Intervention training which adopted both a practical and theoretical approach has been positively evaluated in terms of acceptability and fidelity to the intervention in previous studies [42–45] and in complex interventions for older people [46]. Improvements to the intervention for future studies could include supplemental online learning packages, embracing the growth of blended learning in undergraduate and post-graduate training.

Patient and family participants positively evaluated the care they received but were unable to isolate RePAT from usual clinical care. Older people may face difficulties when recalling events in the hospital setting and expressing them in a formal interview setting [47, 48]. RePAT was completed using information from multiple resources; clinicians did not ‘sit down’ with the patient and complete it. This represents the reality of clinical practice where clinicians assimilate information from multiple sources; much of this is ‘hidden’ from the patient, but its principles should be communicated as part of the ongoing therapeutic relationship.

Thirteen out of fifteen RePAT items achieved an *a priori* fidelity rating of 80% or more. Items which explored underlying and unresolved physical and psychological issues scored 100% fidelity which are components of usual rehabilitation practice [49]. Low fidelity was found in the goal setting component of RePAT. Goals were typically framed from a clinicians or organisational perspective, as opposed to person-centred. Although goal setting has long been a central component of rehabilitation [50] there remains a lack of empirical evidence on the best way of operationalising goals in clinical practice [51] and its suitability for frail older people. Leach et al. [52] explored goal setting in the subacute geriatric rehabilitation setting in Australia. Although specific to the stroke population, they found that patients were not able to fully participate in goal setting and therapists often led or controlled the process with a strong bias towards impairment and activity limitations. So while goal setting seeks to encourage a patient-centred approach to care, the environmental and organisational constraints of acute care and constructs of ill health may weaken this effect. Factors around participation and motivation can lead to a high degree of subjectivity and this may lead to limitations in their applicability.

### Strengths and limitations

An overall limitation of the study remains the lack of a clear primary outcome measure which quantified rehabilitation potential. This may be explained by the differing notions of rehabilitation intervention success, i.e. at individual patient, clinician or organisation level. Further research is required to explore the link between rehabilitation potential and how its outcome is measured and linked to primary research outcomes for future definitive trials. Interviews were completed by the researcher who had designed and delivered the RePAT intervention training. This may have led to social desirability bias with participants positively evaluating the intervention. The sample size was adequate to draw conclusions on deliverability and acceptability but participants were recruited from one site so the findings cannot be generalised.

Fidelity considers if the intervention was delivered as intended and is central in understanding if staff participants were able to deliver the intervention effectively [27] and under what circumstances. This study included measures of fidelity as part of the *a priori* criteria for success.

The study sample was less frail than in other similar studies [53]. The assumption that those with higher degrees of frailty do not respond to rehabilitation has been recently challenged [10, 54–56], however individuals may require more support and time to reach their potential.

*A priori* criteria for success were developed to provide information on whether the study was successful. Staff and patient participants were retained in the study. Patient participants with cognitive impairments, including 31% whom lacked capacity, were recruited into the study using consultee agreement and a form of process consent [57, 58]. They are frequent users of acute healthcare [59]. Cognitive functioning, delirium and mental health issues are widely recognised as factors that adversely affect rehabilitation outcomes amongst older people [60–62] and any tool which did not address these needs would not be fit for purpose. The aim of this study was to identify patients who were admitted for acute general medical care, which excluded femoral fractures and stroke as a primary diagnosis. Participants were most commonly admitted with falls, mobility difficulties and disorientation with a mean number of ICD codes of 2.12 (admitting complaints), representing the heterogeneous nature of presenting conditions and comorbidities of this population [53, 63].

## Conclusion

 The Rehabilitation Potential Assessment Tool (RePAT) was found to be acceptable and feasible to be delivered in the acute hospital setting by patient, staff and carer participants. Physiotherapy and occupational therapy participants reported RePAT enhanced their understanding of rehabilitation potential. It allowed them to make explicit their clinical reasoning behind rehabilitation decision-making and take into account the dynamic nature of frailty and acute ill health. The structure and content encouraged clinicians to become more cognisant of ethical dilemmas and biases in their practice. The tool was completed alongside usual clinical care in a timely manner and with a high level of fidelity. Patients and carer participants, although unable to comment directly on RePAT, reported activities that were most likely as a direct result of the tool being used in the study and embracing a person-centred approach. The next step is to further refine the RePAT intervention based on the findings of this study and test the effectiveness of RePAT in predicting rehabilitation success.

## Supplementary Information

**Additional file 1.** 

## Data Availability

The data that support the findings of this study are available from University of Nottingham but restrictions apply to the availability of these dat. Data are however available from the authors upon reasonable request and with permission of the University of Nottingham.

## Abbreviations

ADL: Activities of Daily Living
AFC: Agenda for Change
AMT: Abbreviated Mental test
CCI: Charlson Co-morbidity Index
HCOP: Health care of older person’s
MDT: Multi-disciplinary Team
MMSE: Mini-mental state examination
OT: Occupational Therapy
RePAT: Rehabilitation Potential Assessment Tool
