# Supplementary material for: A non-randomised feasibility study of the Rehabilitation Potential Assessment Tool (RePAT) in frail older people in the acute healthcare setting
Source: BMC Geriatr. 2022 Oct 7;22:785. doi: 10.1186/s12877-022-03420-w (PMC9541000; doi:10.1186/s12877-022-03420-w)
Supplement: Supplementary file 1 — Additional file 1. [file 12877_2022_3420_MOESM1_ESM.pdf]

Supplementary file one

Rehabilitation Potential Assessment Tool (RePAT)

Rehabilitation potential is a process which involves complex clinical judgement and prognostication of the projected benefits of undertaking a targeted programme of rehabilitation. The assessment of rehabilitation potential should take into account physical and psychological factors identified during multidisciplinary assessments along with individual patient needs and wants and the availability of family support. It involves developing an understanding of who will participate with rehabilitation, in and outside the therapy setting, who can support this and who is likely to benefit.

**Question**	**Comments**
1. Are there any underlying physical issues which may affect or interfere with rehabilitation?	
2. Are there any unresolved physical issues which may affect or interfere with rehabilitation?	
3. Are there any underlying psychological issues which may affect an individual's motivation or participation with rehabilitation?	
4. Are there any unresolved psychological issues which may affect an individual's motivation or participation with rehabilitation?	
5. Has the individual been able to demonstrate participation in the rehabilitation assessment or rehabilitation programme during this current episode of care?	
6. What are the individual's current functional abilities and levels of independence?	
7. What were the individual's pre-morbid (pre-admission) functional abilities and levels of independence?	

Question	Comments
8. Do you have a thorough understanding of individual's environment in their usual place of residence?	
9. What support does the individual require to make decisions about their future?	
10. Has the individual been asked "What's important to me?"	
11. Have goals been set and agreed which are SMART? (Short, Medium, Long Term)	
12. Has the multi-disciplinary team been involved in the assessment or decision-making process?	
13. Has the individual's rehabilitation potential been assessed over multiple time points?	
14. Is the proposed rehabilitation programme likely to be effective?	

Overall impression

Time taken to complete assessment=
Time taken to complete form =

Guidelines for completing the tool

1. Consider the impact that any underlying physical issues or medical conditions might have on an individual's ability to benefit from or participate in rehabilitation. Are these being actively managed, can they be optimised, are they stable or unstable, reversible or irreversible? Are you able to predict their trajectory or recovery? For example, severe cardiorespiratory conditions or advanced heart failure, frailty syndrome, co-morbidities or poor nutritional status.
2. Consider the impact that any unresolved physical issues or acute events (such as an infection, fall or fract ure) might have on an individual's ability to benefit from or participate in rehabilitation. Are these being actively managed; are they stable or unstable, reversible or irreversible? Are you able to predict their trajectory or recovery? For example, ongoing treatment for an infection, poor nutritional status, pain or heart failure.
3. Consider underlying psychological issues which may impact on an individual's ability to participate or benefit from rehabilitation. Are these new or ongoing issues? What management strategies have or could be put in place to enable the individual to receive and be an active partner in rehabilitation? For example; antidepressant medications for low mood, delirium, memory issues, and disruptive behaviours for which strategies or specialist teams may be advised. Consider if these issues are exacerbated by an individual's acute illness, underlying conditions or the hospital environment.
4. Consider unresolved psychological issues which may impact on an individual's ability to participate or benefit from rehabilitation. Are these new or ongoing issues? For example a diagnosed or undiagnosed delirium. What management strategies have or could be put in place to enable an individual to receive rehabilitation? For example; antidepressant medications for low mood, delirium, memory issues, grief, and disruptive behaviours for which strategies or specialist teams may be advised. Consider if these issues are exacerbated by their acute illness, underlying conditions or the hospital environment.
5. During the assessment, rehabilitation or routine care, has the individual been able to participate? Do they require verbal or non-verbal prompts? Is their participation consistent or does it fluctuate? Consider the impact that medical and psychological factors can have on participation. Do they take an interest in the assessment or rehabilitation? Motivation and participation may fluctuate and differ once the individual has recovered from an acute illness, delirium or returns to their usual place of residence. If they have previously been in receipt of rehabilitation consider contacting community services to determine previous rehabilitation response and/or participation.
6. Consider an individual's current functional abilities and levels of independence. What is the individual able to do in the hospital setting either with or without support? This may include transfers, mobility, self-care activities. When assessing for current abilities be aware of the limitations which the hospital environment, their underlying conditions or acute event may have on the patient. For example; reduced mobility or exercise tolerance due to admission. Understanding these factors may have an impact on which future rehabilitation services you decide to refer to.
7. Consider the individuals pre-morbid or pre-admission levels of function and independence. What was the individual's previous level of functional abilities in the days, weeks and months prior to this hospital admission? Supplement this with information from family members, carers and community service providers. Ensure that you have a good understanding of the impact that underlying medical conditions and psychological issues have on pre-morbid abilities and their trajectories.
8. What type of accommodation do they usually reside in? Consider existing aids, adaptations, access and what the individual needs to be able to do to return there. If you are considering referral to inpatient or community residential rehabilitation ensure that you have a good understanding of this environment and its suitability for the individual.
9. Does the individual have capacity and/or insight to make decisions about their future needs, care and rehabilitation choices? What support does the individual require to make these decisions? Is an assessment of Mental Capacity Required? Who is able to support the individual in this process; family, carers, GP? Bear in mind that capacity may fluctuate in the hospital setting or with acute ill health and conditions such as delirium.

10. Ask the individual "what is important to me" to establish what they want to achieve or value. In the hospital setting this may include returning to their usual place of residence, personal relationships or activity of daily living. Where individuals may lack capacity or cognitive function, family and friends may be able to provide information.
11. Goals which are Specific Measureable Achievable Realistic and Timed (SMART) should be discussed and agreed with the individual based on "What is important to me". Short term goals may include leaving hospital care. Medium term; independence with mobility, self-care. Long term; outdoor mobility, social interactions. Be aware that goals set in hospital may change once the individual has been transferred or returning to their usual place of residence. Goals should focus on the individual and not what the therapist wants them to achieve.
12. Incorporate assessment findings from other members of the multi-disciplinary team. Use verbal or written information to build up a picture of the individual's needs, wants and abilities. For example; nurses, doctors, social workers, psychologists, family, friends and carers. Community based staff such as social care support workers, community therapists and GP's should be consulted. Nursing staff are ideally placed to provide a 24 hour view of an individual's abilities, levels of motivation and participation in the hospital setting.
13. An individual's needs and abilities should be assessed more than once. This may either be through direct assessment, observation or other team member's assessments. If you are unable to establish this in the hospital setting consider referring for a community follow up assessment. This is especially important in the acute setting when an individual may be unwell, recovering from a period of ill-health, sleep deprived or in an unfamiliar environment. This should be considered over a 24 hour period.
14. Consider if the proposed rehabilitation programme is likely to be effective. Take into account an individual's likely response and predicted benefit. Do you have a good understanding of the evidence base on the effectiveness of rehabilitation in this patient population? Are you able to predict likely response?

Overall impression

Use this space to reflect on your overall impression of the individuals' rehabilitation potential, detailing your clinical reasoning and thought process. You may want to use this space to consider future plans for the patient.
